# Choice-Supportive Misremembering: A New Taxonomy and Review

**DOI:** 10.3389/fpsyg.2017.02062

**Published:** 2017-12-04

**Authors:** Martina Lind, Mimì Visentini, Timo Mäntylä, Fabio Del Missier

**Affiliations:** ^1^Department of Psychology and Cognitive Science, University of Trento, Trento, Italy; ^2^Department of Life Sciences, University of Trieste, Trieste, Italy; ^3^Department of Psychology, Stockholm University, Stockholm, Sweden

**Keywords:** decision making, episodic memory, choice-supportive memory, positivity bias, misattribution, fact distortion, false memory, selective forgetting

## Abstract

Although the literature on the influence of memory on decisions is well developed, research on the effects of decision making on memory is rather sparse and scattered. Choice-supportive misremembering (i.e., misremembering choice-related information that boosts the chosen option and/or demotes the foregone options) has been observed in several studies and has the potential to affect future choices. Nonetheless, no attempt has been made to review the relevant literature, categorize the different types of choice-supportive misremembering observed, and critically appraise the existing evidence and proposed explanations. Thus, starting from a new theoretically motivated and empirically grounded taxonomy, we review the current research. Our taxonomy classifies choice-supportive misremembering into four conceptually distinct types: *misattribution* is when information is attributed to the wrong source, *fact distortion* when the facts are remembered in a distorted manner, *false memory* when items that were not part of the original decision scenarios are remembered as presented and, finally, *selective forgetting* is when information is selectively forgotten. After assessing the impact of various potentially moderating factors, we evaluate the evidence for each type of misremembering and conclude that the support for the phenomenon is solid in relation to misattribution when recognition memory is assessed, but significantly weaker for the other three types, and when other memory tests are used to assess memory. Finally, we review the cognitive and emotional explanations proposed for choice-supportive misremembering in the light of the available evidence and identify the main gaps in the current knowledge and the more promising avenues for future research.

## Introduction

Time and memory are true artists; they remold reality nearer to the heart’s desire

(John Dewey, Reconstruction in Philosophy, 1950).

Decision-making processes have been widely studied both in basic research and in applied contexts, with a significant part of recent research concerning the impact of memory processes and memory-related biases on decisions (see e.g., Dougherty et al., 2003; Tomlinson et al., 2011; Del Missier et al., 2013, 2015; Hoffmann et al., 2014). Despite this interest in the relationships between memory and decision making, only a limited number of studies have investigated how decision making affects memory, and, more specifically, how the act of choosing and the actual choice one has made influence subsequent memory of the options (e.g., Mather and Johnson, 2000; Mather et al., 2000, 2003). Although the issue of choice-supportive misremembering has both theoretical interest for cognitive and decision scientists and applied implications for practitioners in a variety of fields, research has been sparse and non-systematic and a unifying review is currently lacking. Moreover, each relevant paper tends to focus mainly on one of the different types of misremembering, but there has been no overarching attempt to clarify the categories into which such systematic distortions may be divided and their relationships. Finally, for some of these effects, results are not fully consistent across studies and diverse explanations have been proposed.

Given this state of affairs, the present review has two aims: (1) to introduce a new taxonomy useful for understanding choice-supportive memory effects and their underlying processes; (2) to review the literature on choice-supportive memory and appraise the degree of support for the different aspects of the phenomenon and for the existing explanations. We will start by proposing a new theoretically motivated and empirically grounded taxonomy describing the possible types of systematic choice-supportive misremembering that decision making may induce. Then, we will review papers accounting for the influence of potentially moderating factors on choice-related misremembering after decision making (i.e., alignability of attributes, delay before memory test, valence of stimuli, individual differences, and type of memory test). After that, following our taxonomy, we will appraise whether choice-supportive misremembering is a robust and well-supported phenomenon both within each category and overall. We will also discuss the proposed explanations for choice-related misremembering in terms of underlying cognitive and affective processes.

From the theoretical viewpoint, the novel taxonomy and the associated review offer a new unifying and clarifying perspective on rather disconnected effects and phenomena, and the potential reasons behind them. This will highlight the similarities and differences between various kinds of misremembering after choice, allow an appraisal of their respective degree of empirical support, and provide more insight into the underlying processes. Furthermore, it will shed light on underinvestigated aspects, unresolved issues, and the more promising new research directions. From the applied research viewpoint, gaining insight into whether, when, and why decision-making processes distort our memory could eventually help us determine to what extent human memory can be trusted and give indications on how to improve memory-based decision making. Indeed, a strongly altered memory of past choices may affect future choices and hinder proper learning from experience and adaptation to reality.

### A New Taxonomy of Misremembering after Decision Making

**FIGURE 1:**
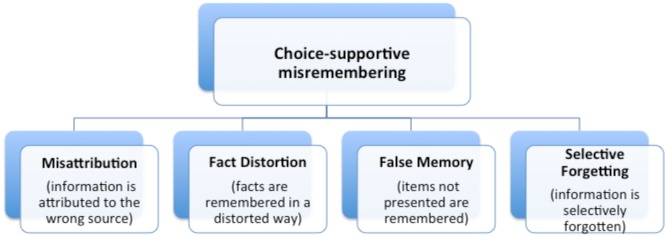
The taxonomy of choice-supportive misremembering.

Starting from a theoretical analysis and a review of the literature connecting memory and decision making, we propose a new taxonomy and analysis systematically addressing choice-supportive misremembering after decision making (i.e., misremembering choice-related information in a way that boosts the chosen option and/or demotes the foregone options). We identified four conceptually distinct types of choice supportive misremembering, with clear face validity, corresponding to diverse research streams in the decision-making and memory literatures: *misattribution, fact distortion, false memory*, and *selective forgetting* (**Figure 1**).

Support for the proposed taxonomy comes from three different sources: (1) the a-priori grounding of the taxonomy categories in diverse and complementary theoretical views, (2) the face validity of the different types of misremembering that can be logically disentangled, (3) the empirical support coming from the studies that will be reviewed in the present paper. We introduce the taxonomy before presenting the review in order to provide a clear organizing principle for the description of the studies.

Misattribution is when positive attributes are remembered as belonging to the chosen option when they in fact belonged to the foregone option, or when negative attributes are remembered as belonging to the foregone option when they in fact belonged to the chosen option. For example, if the choice is between two houses and the chosen house has a hole in the roof and the foregone one has a wonderful view, the chosen house is remembered as having a wonderful view or the foregone one as having a hole in the roof. Misattribution is a well-known type of commission error, where a memory is misattributed to the wrong source, and one of Schacter’s “seven sins of memory” (Schacter, 1999). In line with research in the decision-making field (Mather and Johnson, 2000; Mather et al., 2003), however, our operational definition is narrower than Schacter’s, specifically referring to the attribution of a correctly recalled feature to the wrong option. Indeed, misattribution presupposes correct encoding and recall of the actual information – only its source is confused – while false memory represents a separate category in our taxonomy, since it is a qualitatively different type of error (remembering information never presented), which may even be related to different underlying processes (e.g., Reyna and Lloyd, 1997), an issue that will be discussed later in the paper.

Fact distortion is when the objective values of features belonging to the chosen option are misremembered as more preferential than their actual values, and values of features belonging to the foregone option as less preferential. An example would be if in the choice between the two houses both were located 1 km from the work place, but the chosen one is remembered as being 500 m away and/or the foregone one as being 1.5 km away. This actual distortion of facts is distinct from changes in the subjective evaluation or attractiveness of options during the decision-making process, which are widely studied phenomena in the decision-making literature (e.g., Svenson and Benthorn, 1992; Russo et al., 1996; Holyoak and Simon, 1999; Shamoun and Svenson, 2002). Altering specific pieces of information is an error of commission possibly related to the biasing influence of current beliefs on memory (Schacter, 1999). Fact distortion in memory after choice has been specifically postulated by the differentiation-consolidation theory (Svenson, 1992) and investigated by Svenson et al. (2009) and DeKay et al. (2014) in studies that will be discussed later in this review.

False memory in the context of choice-supportiveness is when new attributes that were not part of the original options are ‘remembered’ as presented and, if their values are considered positive, as belonging to the chosen option, and if negative, as belonging to the foregone option. For example, the chosen house might be remembered as being well insulated even though no information about the insulation of either house had been presented. False memories have been widely investigated in the memory literature, for instance in relation to the misinformation paradigm (e.g., Ayers and Reder, 1998; Laney et al., 2008; Bernstein and Loftus, 2009; Cochran et al., 2016). Some research has also been carried out on false memories in relation to decision making (e.g., Pennington and Hastie, 1988; Sharman et al., 2008; Lindholm et al., 2014; Corbin et al., 2015). They represent a more dramatic and radical departure from reality than simple fact distortions, in that an entire new piece of non-existing evidence is remembered. Moreover, this type of error is not attributable to a properly encoded but later confused feature, thus it is conceptually distinct from misattributing a correctly recalled feature to one of the presented options (for a classification of memory errors and false memory phenomena see also Reyna and Lloyd, 1997).

Selective forgetting is when the positive attributes of the chosen option and the negative attributes of the foregone option are remembered at a higher rate than vice versa. An example would be correctly remembering that the chosen house was close to the work place, but forgetting its leaking roof. This is a typical omission error, possibly fostered by the decreasing accessibility of memory over time (“transience”: Schacter, 1999; Schacter et al., 2003). Selective forgetting and remembering has traditionally been studied in relation to the confirmation bias (e.g., Levine and Murphy, 1943; Nickerson, 1998). Mather et al. (2000) and Depping and Freund (2013) have investigated the occurrence of this phenomenon after choice. Moreover, selective forgetting has been studied more generally as the outcome of incidental or motivated forgetting processes in the memory literature (e.g., Bäuml, 2008; Anderson and Huddleston, 2012; Hirst and Echterhoff, 2012; Anderson and Hanslmayr, 2014).^1^

### Eligibility Criteria for the Review

We included in the review only studies in which participants were presented with at least two options with multiple features, thus focusing on traditional multi-attribute choice problems, which represent the typical scenarios investigated in decision-making (e.g., Payne et al., 1993). Moreover, in the selected studies, participants were asked to make a deliberate preferential choice between the options after reviewing these features, which qualifies only proper decision-making studies (e.g., Hastie and Dawes, 2010). Based on evidence of important differences between judgment and choice processes (see e.g., Payne et al., 1993; Lichtenstein and Slovic, 2006), any study where the participants are asked to make a judgment rather than a choice (e.g., Dellarosa and Bourne, 1984; Lindholm et al., 2014) or the decision is based on a mere esthetic preference (e.g., Lieberman et al., 2001; Johansson et al., 2005) was also excluded from this review.

In addition to these structural selection criteria, which adhere to traditional distinctions in judgment and decision-making research, we adopted some additional criteria with the specific aim to exert more control over the possibility that the conclusions drawn from the literature review are actually attributable to the influence of choice making on memory processes and not affected by extraneous factors. In particular, we excluded studies in which additional information (including misinformation) was introduced between the choice and the memory test. This left out from the review research on other types of distortions, such as those resulting from well-known hindsight paradigms. Indeed, in these conceptually different types of situations, the memory distortion is at least partly produced by the provision of information after the decision or the experience and not by the decision *per se* (for reviews see e.g., Erdfelder et al., 2007; Louie et al., 2007; Bernstein et al., 2011; Calvillo, 2012). Furthermore, in this review we are concerned solely with preferential choice and not with the predictability of an event or outcome, which is the main issue investigated in studies on the hindsight bias (Roese and Vohs, 2012). We also excluded studies in which participants themselves select which information to access (as in, e.g., Redlawsk, 2001; Meffert et al., 2006).

Finally, the memory test had to be of the information provided and not of the influence of prior knowledge (see e.g., Dellarosa and Bourne, 1984; Biehal and Chakravarti, 1986) or merely of the choice made (e.g., Holyoak and Simon, 1999; Hall et al., 2010), because these latter tests do not allow investigating choice-supportive misremembering.

One study that appeared to qualify was excluded on the basis of lack of details (Davidson and Kiesler, 1964), since our attempt to obtain more information from the authors was unsuccessful. Another study with partial information was included (Chen and Zhang, 2003) but, due to the lack of detail, our review of that study is limited.

Thorough searches using the key words “memor^∗^,” “recall^∗^,” “recog^∗^,” “remem^∗^,” and “recoll^∗^” combined with “decision,” “choice,” “option,” and “prefer^∗^” were conducted in the databases ERIC, Psycarticles and PsycINFO, as well as in Google Scholar. Additionally, after assessing the eligibility of the articles or proceedings found, all backward and forward references of the eligible papers were assessed using both Web of Science and Scopus. The time period covered by the search was until July 2017. Postings were made to relevant decision-making mailing lists (Society of Judgment and Decision Making, and European Association for Judgment and Decision Making) asking for both published and unpublished papers on memory biases or distortions or misremembering after choice. **Table 1** summarizes the main features of the studies satisfying our inclusion criteria.

**Table 1:** Summary of the eligible studies.

**Study**	**Main misremembering category**	**Participants (age)**	**Conditions or groups**	**Scenarios**	**Attribute alignability**	**Delay**	**Memory test**
	*[other categories potentially relevant]*						

Benney and Henkel, 2006	Misattribution *[Selective forgetting, False memory]*	172 adults (18–52)	Best interest vs. free choice vs. assigned option	• Restaurants	Mainly unalignable, but gum options partly alignable	30 min	Recognition (source recognition)
				• Movie theaters			
				• Department stores			
				• Gum			
Chen and Zhang, 2003	Misattribution *[Selective forgetting]*	Number and age of participants not known	High vs. low conflict scenarios Delay levels	Not known	Not known	“Short” and “long” (details not known)	Free recall and source recognition (details not known)
• Experiment 1							
• Experiment 2							
DeKay et al., 2014	Fact distortion	• Experiment 1: 169 adults (18–68)	Choice vs. no choice	• Apartments	All alignable	No delay	Recognition (forced-choice recognition)
• Experiment 1		• Experiment 2: 470 adults (18–71)					
• Experiment 2		• Experiment 4: 255 adults (18–74)					
• Experiment 4							
Depping and Freund, 2013	Selective forgetting *[Misattribution]*	• Experiment 1: 66 young (19–30), 73 older (60–88)	Choice vs. readability (no choice) Age	• Travel packages	Mainly not alignable	7 min	Free recall
• Experiment 1		• Experiment 2: 62 young (18–31), 60 older (64–86)		• Hospitals (for surgery)			
• Experiment 2							
Henkel and Mather, 2007	Misattribution *[Selective forgetting, False memory]*	80 young adults (18–24)		• Roommates	Mainly not alignable	2 days	Recognition (source recognition)
• Experiment 1				• Internships			
				• Apartments			
				• Cars			
				• Dating partners			
Hess and Kotter-Grühn, 2011	Misattribution *[Selective forgetting]*	54 young (20–44), 52 middle-age (45–64), 54 older (65–85)	Impression (no choice) vs. interaction (and choice) Age	• Persons with whom to spend a day (social partner)	Not known, but the examples provided in the paper suggest mainly unalignable	Short, length not specified	Recognition (source recognition)
• Experiment 1							
Hess et al., 2012	Misattribution	54 young (20–44), 55 middle age (45–64), 54 older (65–85)	Active deliberation vs. no deliberation Alignable vs. unalignable attribute focus Age	• Grocery store	Half alignable	Relatively short, length not specified	Recognition (source recognition)
				• Apartment to rent			
Mather and Johnson, 2000	Misattribution *[Selective forgetting, False memory]*	54 young (18–26), 108 older (64–83)	Affective review vs. factual review vs. no review Delay levels Age	• Houses	Mainly not alignable	30 min, 2 days	Recognition (source recognition)
				• Job candidates			
				• Flights			
				• Blind dates			
Mather et al., 2000	Misattribution *[Selective forgetting, False memory]*	• Experiment 1: 142 students	Experiment 3b: Choice vs. rejection	• Job candidates	Mainly not alignable	Experiment 1: 5 min Experiments 2 and 3a: 45 min Experiment 3b: 5 min	Recognition (source recognition)
• Experiment 1		• Experiment 2: 75 under-graduates		• Blind dates			
• Experiment 2		• Experiment 3a: 77 undergraduates		• Roommate			
• Experiment 3a		• Experiment 3b: 379 students					
• Experiment 3b							
Mather et al., 2003	Misattribution *[Selective forgetting, False memory]*	94 undergraduates (age not known)	Choice vs. assignment	• Houses	Mainly not alignable	45 min	Recognition (source recognition)
• Experiment 2				• Roommates			
				• Cars			
Queen and Hess, 2010	Misattribution *[Selective forgetting]*	62 young adults (17–28), 75 older adults (60–86)	Age Conscious vs. unconscious thought Deliberative vs. Intuitive information	• Apartments	All attributes alignable	Relatively short, not specified	Recognition (source recognition)
				• Banks			
Svenson et al., 2009	Fact distortion	• Experiment 1: 64 students (21–42)		Patients needing surgery	All attributes alignable	Experiments 1 and 2: Not known Experiment 3: 1 h	Cued recall
• Experiment 1		• Experiment 2: 35 students familiar with scenario type (23–48),					
• Experiment 2		• Experiment 3: 77 students (19–39)					
• Experiment 3							


*^a^Participants included in the analyses.*

*^b^The age of the participants not specified for any of the experiments in this paper.*

### Influence of Choice on Choice-Supportive Misremembering

Before discussing research on choice-supportive misremembering, it is necessary to appraise whether a necessary condition for the existence of this phenomenon holds: the very act of making a choice should influence subsequent memory of the options. Thus, it is essential to assess whether the effects are only observed after choice or whether they are also found when no active choice has been made. For this reason, several of the experiments use a design with a control group whose participants do not make a choice but are simply assigned an option (Mather et al., 2003; Benney and Henkel, 2006; Hess and Kotter-Grühn, 2011). In other studies participants are provided a “best interest” option (Benney and Henkel, 2006), or asked to focus on “readability” of a text rather than its contents (Depping and Freund, 2013).

All of these studies, apart from the latter, found that making a choice induces choice-supportive misremembering. Such misremembering was found for assigned options only when the participants had been led to believe that the assignment was based on their best interest (Benney and Henkel, 2006). When participants had not been told that the assignment was in their best interest, participants’ memory slightly favored the option they had not been assigned. In particular, Benney and Henkel did not observe choice-supportive misremembering in the assignment group and they observed the effect both in the free choice group and the best interest group, with these two latter groups not differing significantly.

Likewise, Mather et al. (2003) found choice-supportiveness in their choice group, but not in the assignment group. Hess and Kotter-Grühn (2011) also found significantly higher level of choice-supportiveness of memory in the social interaction (choice) condition than in the impression (no choice) condition, but as the participants in the impression group were not told which option they had been assigned, the design did not enable an analysis of the effect on memory of being assigned an option. It is also worth noting that Mather et al. (2000) observed similar choice-supportiveness when participants had to choose one option vs. when they had to reject one option. Interestingly, Henkel and Mather (2007) observed misattribution to be supportive of the choice they thought they had made rather than of their actual choice. As pointed out by the authors, this indicates that belief at the time of retrieval can influence memory accuracy and thus that the observed misremembering cannot be entirely due to encoding processes. Only Depping and Freund (2013) did not find choice-supportive memory in the groups making a choice.

## Factors Potentially Influencing Memory Choice-Supportiveness

Before reviewing the studies within each category of choice-supporting memory, we will discuss some factors potentially moderating misremembering, given that these factors may be influential across the four proposed categories. The main potential moderating factors are alignability of the attribute values, delay between presentation of the options and memory test, valence (of the scenarios, options and attributes), individual differences, and type of memory test. These factors have been found to affect decision making or memory significantly (e.g., Payne et al., 1993; Markman and Medin, 1995; Schacter and Coyle, 1997; Schacter, 1999) and thus they may play also a role in choice-supportive misremembering.

### Alignability

A factor that may influence misremembering after choice is alignability (i.e., whether both options have directly comparable features). For example, when choosing between houses, an attribute value belonging to one option may be that it is 1 km from the city center and one belonging to the other option that it is located 2 km from the city center. These attribute values are alignable (or commensurable), whereas those features that do not have a comparable one in the other option are not.

The effects of alignability on decision making or memory have been investigated in a number of studies (e.g., Markman and Medin, 1995; Markman and Gentner, 1997; Mather et al., 2005), and the degree of alignability varies across the studies we reviewed. All attributes of the options in Svenson et al. (2009), Queen and Hess (2010) and DeKay et al. (2014) were alignable, whereas others employed a design where half of the attributes were alignable and the other half unalignable (Budson et al., 2006; Hess et al., 2012). The remaining studies did not specifically assess the importance of alignability and did not specify the alignability of the attributes. From the evidence available, it appears that none of the remaining papers used scenario features that were entirely or substantially alignable.

Choice-supportive memory has been observed both in studies with alignable attributes and in studies with non-alignable attributes (see **Table 1**). However, Hess et al. (2012) observed an interaction between age and alignability, with choice supportiveness scores increasing from the young to middle-aged to older groups for alignable attributes but not for unalignable attributes. This effect was no longer significant when a composite ability measure was used as a covariate. Generally, they observed greater choice-supportiveness for alignable attributes. This suggests that the effect can be greater for alignable features, at least in some populations (see also Mather et al., 2005; Budson et al., 2006), and that this difference can be related to cognitive skills. However, more studies are needed to fully clarify the role of alignability in choice-supportive misremembering, especially in relation to the category of false memories and in different populations.

### Delay

An important factor affecting memory is the extent of the delay between encoding (i.e., viewing the information about the options) and retrieval (i.e., the memory test). An increased delay is likely to result in more misremembering, but it is possible that delay affects different types of memory distortions differently. Unfortunately, although some memory studies have found false memories to increase over time (e.g., Sulin and Dooling, 1974; Howe et al., 2010), the relative influence of delay, memory test, and material used on choice-supportive misremembering has not been properly scrutinized. Indeed, none of the studies reviewed allow us to draw strong conclusions about this, as they did not systematically investigate types of misremembering vs. delay. However, some used a design with different delay levels in the same study (Mather and Johnson, 2000; Chen and Zhang, 2003), showing how delay affected the particular type of memory distortion studied. The other experiments all used rather short but variable time lags (Budson et al., 2006; DeKay et al., 2014).

Chen and Zhang (2003) found that when using a “long” delay, positive attributes were more likely to be attributed to the chosen option in their high-conflict condition than in their low conflict condition (similar vs. diverse attractiveness of the choice options). Unfortunately, that paper does not specify exactly the length of the delays used, and we have not been able to obtain more information about the experiment from the authors. Mather and Johnson (2000) found that, although most participants exhibited a source attribution bias favoring their chosen option, there was no significant effect of delay (30 min vs. 2 days) other than the group with older participants and the longest delay (2 days) showing the weakest memory for every measure. It is clear that studies further investigating the effect of delay on choice-supportive misremembering are needed. Not only would it be interesting to see whether delays longer than 2 days (the longest delay after which choice-supportiveness was assessed) produce more choice-supportive misremembering compared with shorter delays, but also whether delay specifically affects the different types of distortions observed.

### Sequential vs. Simultaneous Presentation of Information

Choice-supportive memory phenomena can be the product of biased encoding or biased recall of the information presented (or both). In the former case, the decision maker may have encoded altered or partial information before making a choice (predecisional distortion: DeKay, 2015). In the latter case, choice-supportive memory originates from processes occurring after a decision has been made. Sequential presentation of information (vs. simultaneous presentation) might favor predecisional distortion (DeKay et al., 2014).

The studies we reviewed vary in relation to the information presentation type. Some of them used a sequential presentation of the options or attributes (Queen and Hess, 2010; Hess and Kotter-Grühn, 2011; Hess et al., 2012; DeKay et al., 2014), whereas the participants in the remaining studies made their choice with the information simultaneously and externally available. Choice-supportive misremembering has been observed both with sequential and with simultaneous presentation of information. However, given that no study appraised systematically the influence of information presentation on choice-supportive memory, more research is needed.

### Valence

The impact of valence in relation to misremembering after choice can be evaluated at three levels: scenario, option, and attribute. Making a choice in a positive scenario or situation (e.g., going on holiday and choosing between two different destinations) may not result in the same degree and kind of misremembering as a choice in a negative scenario or situation (e.g., being seriously ill and choosing between two different methods of surgery). Similarly, a desirable and an undesirable option, or positive and negative attributes, may be distorted to different degrees. Out of the studies reviewed, only Depping and Freund (2013) attempted to clarify whether the valence of the scenarios/situation influence memory distortion. They conducted two experiments with older and younger participants to investigate the impact of valence and choice on memory. Unfortunately, Depping and Freund failed to observe significant choice-supportive misremembering in their studies, regardless of the age group investigated, and therefore no interaction with valence was detectable. However, the other studies we reviewed found significant choice-supportive memory using positively, neutrally and negatively valenced settings, although only Svenson et al. (2009) used a clearly negative scenario. This implies that valence may not be critical for observing choice-supportive memory effects, although more studies systematically investigating its influence on choice-supportive misremembering are needed.

### Individual Differences

Not everyone may misremember to the same degree. Age as well as individual differences in terms of cognitive abilities and personality are likely to be influential. Thus, several of the studies included different age groups (Mather and Johnson, 2000; Mather et al., 2005; Queen and Hess, 2010; Hess and Kotter-Grühn, 2011; Hess et al., 2012; Depping and Freund, 2013), and some also individual differences in cognitive ability (Mather and Johnson, 2000; Queen and Hess, 2010; Hess and Kotter-Grühn, 2011; Hess et al., 2012).

As far as overall choice-supportiveness is concerned, only Mather and Johnson (2000) found that older age is associated with greater choice-supportive misremembering (in particular, source misattribution). Indeed, Queen and Hess (2010), Hess and Kotter-Grühn (2011), Hess et al. (2012), and Depping and Freund (2013) all observed that the memory of older adults was not significantly more choice-supportive than that of younger adults. Although age has mainly been looked at in relation to valence or overall choice-supportiveness, Hess and Kotter-Grühn (2011) introduced another dimension: morality vs. competence judgments. The participants in their first experiment were older and younger adults, rating target persons based on statements focusing on either morality or competence. The participants in one subgroup made an impression of the target persons and those in the other chose whom to spend a day with socially. Only in the older adult group was choice-supportiveness specific to the morality domain. Curiously, although different adult age groups have been investigated in several studies, research in children is lacking and it would represent an interesting avenue for future investigations.

The participants in three of the reviewed studies (Mather and Johnson, 2000; Hess and Kotter-Grühn, 2011; Hess et al., 2012) completed cognitive tests as part of the experiments. However, only Mather and Johnson (2000) discussed the correlations between the scores obtained there in older participants and the observed choice-supportiveness in memory. They assessed cognitive capacity with nine neuropsychological tests, and found significant correlations between memory choice-supportiveness and scores on tests requiring the kind of executive and reflective processing associated with the frontal lobes of the brain, but no significant correlations with tests of memory functions associated with the medial-temporal regions (only in their control condition). They also did not find correlations between overall memory accuracy and choice-supportive memory. In particular, participants with better performance in tests of frontal/executive functioning were *less* prone to choice-supportive memory.

Mather and Johnson (2000) set out to explore the influence of emotional/motivational factors on memory after decision making by comparing groups of participants assigned to three different review conditions: affective (think about how you felt about the options), factual (review the details of the options) and no review (filler task). The emotional focus in the affective condition increased the rate of choice-supportive memory in younger adults. This was the case despite the fact that the actual choice features were remembered equally well in the different review conditions. Interestingly, general memory capacity and even recognition accuracy in the specific scenario did not predict the level of choice-supportive misattribution.

### Memory Test

Given that “whether a person remembers an event depends on how memory is assessed” (Roediger and Gallo, 2001, p. 19), the method of testing memory is likely to have an impact on memory distortion. In the majority of the reviewed studies, memory of the options was tested through recognition. For instance, after choosing between two houses to purchase (“Red brick house” vs. “White house built of wood”), participants were asked to say whether “Safe neighborhood” was a feature belonging to the former or to the latter option or if was a new feature (never presented) (Mather and Johnson, 2000). Chen and Zhang (2003) and Mather et al. (2005), however, also included free recall (e.g., asking participants to recall all the attributes they could from each of the choice options). Unfortunately, Chen and Zhang (2003) did not mention whether any differences were found between the two assessment methods, and we have not been successful in obtaining more information about their experiment and findings. Mather et al. (2005) did not assess choice-supportive memory in their two free recall experiments. Depping and Freund (2013) used free recall and Svenson et al. (2009) cued recall. No choice supportiveness was observed in the former study, while choice-supportive fact distortion was observed in the latter one.

To sum up, we cannot draw any strong conclusions about the influence of the type of memory test, as none of the papers specifically addressed this question. In particular, although choice-supportive memory has been observed repeatedly with recognition paradigms, it is not known whether the effect is reliable with free recall due to the scarcity of studies. In future research, it would be interesting to decipher whether recognition, cued and free recall yield differences in choice-supportive misremembering, also considering that free recall and cued recall are the more likely situations to occur in real life when someone is trying to remember the features of the options of a past choice in view of a related one.

A more specific issue related to the memory test concerns the fact that recognition tests for source attribution generally include both new and old features, where the old features are those that had been attributed to one option in the initial presentation and the new ones are the foils for the test. According to some scholars, assessing the extent to which old and new features are misattributed is not only useful to unveil choice-supportive misremembering, but it can also give some partial insight into when in the memory process the distortion is likely to have occurred. Benney and Henkel (2006), for example, found more choice-supportive memory for old than for new features. Both correctly and incorrectly, participants were more likely to attribute positive old features to the chosen option, but this was not the case for new features. Conversely, Henkel and Mather (2007) found similar choice-supportive misattribution for old and new features. As they pointed out, although it may be the case that participants are more attentive to the positive features that subsequently make them choose one option over the other, the fact that choice-supportive distortion was observed also for new features indicates that biased encoding alone may not explain the observed systematic memory distortion. Mather et al. (2003) found that positive old features were both correctly and incorrectly more likely to be attributed to the chosen option. Negative old features, on the other hand, were more likely to be attributed to the foregone option, although this effect was significantly smaller. Finally, positive new features were even more likely to be attributed to the chosen option than any of the old attributes, and negative new features were the ones most likely to be attributed to the foregone option. Similarly, Mather et al. (2000) found choice-supportive memory for old and new features, although it did not reach significance for new features in half of their scenarios. Again, the largest choice-supportiveness effect was found from positive features attributed to the chosen option rather than negative features attributed to the foregone option.

### Other Factors

Other factors could also affect misremembering after choice. For example, Queen and Hess (2010) and Hess et al. (2012) investigated the impact of deliberation (as opposed to intuition) during decision making on choice and on subsequent memory. Whereas Queen and Hess (2010) found no effect, Hess et al. (2012) observed more choice-supportive memory with no deliberation than with active deliberation, which was in line with their hypothesis that more attentive processing would decrease choice-supportive misremembering.

Svenson et al. (2009) found that memory distortion effects were stronger on conflicting attributes than on the attribute that turned out to be the most decisive. One of the hypotheses supported in their experiments was that there would be no consolidation of the attribute that each participant considered to be the most important. This provides some support for the notion that a higher degree of conflict on less important attributes would increase the memory distortion of the attributes. Chen and Zhang (2003), who focused on the differences in memory distortion between high and low conflict options, also found more choice-supportive memory where the options were more balanced in terms of attractiveness (high conflict) when testing the participants after a “long” delay (the precise length of which is unspecified).

Another factor that was looked at in one of the studies reviewed was how beliefs of what choice one has made affects subsequent memory (Henkel and Mather, 2007). Here, it was found that memory was biased in favor of the options the participants thought they had selected rather than their actual choices. The only difference compared to correctly remembered choices was that only somewhat, but not significantly, more negative items were attributed to the option they believe they had rejected. Not surprisingly, source accuracy was superior when the choice was correctly remembered. Similarly, Mather and Johnson (2000) discovered that, with increased delay, the believed choice became more likely to impact memory attributions than the actual choice. As the authors point out, this indicates that beliefs held at the time of retrieval is sufficient to create memory distortion, thus pointing to a crucial influence of the retrieval/test stage in the generation of choice-supportiveness.

## Choice-Supportive Misremembering

### Misattribution

The first type of memory distortion after decision making, misattribution, can be described as a type of choice-supportive misremembering where experimenters observe that participants misattribute attribute values to the wrong option when their memory is being tested after a delay. As mentioned previously, this is a narrower and thus more precise definition than previous ones (e.g., Johnson et al., 1993; Schacter and Coyle, 1997). Misattribution is the most widely studied phenomenon in relation to memory distortion after choice. Much of the body of research on choice-blindness also investigates a kind of misattribution phenomenon of identifying a foregone option as the chosen one after a short delay (e.g., Pärnamets et al., 2015; Somerville and McGowan, 2016) as do other studies on the effect of bias on memory (Frost et al., 2015).

Several studies found choice-supportive misattribution (Mather and Johnson, 2000; Mather et al., 2000, 2003; Chen and Zhang, 2003; Benney and Henkel, 2006; Henkel and Mather, 2007; Queen and Hess, 2010; Hess and Kotter-Grühn, 2011; Hess et al., 2012), and none of the studies in this category that investigated choice-supportiveness failed to find this effect. From the reviewed studies, as we have already seen in the *Memory Test* section, it can also be concluded that the choice-supportive memory is more due to attributing positive features to the chosen option than to attributing negative items to the foregone option, although both of these phenomena are common.

Several processes may underlie misattribution. Biased encoding, errors in source attribution, and reconstructive remembering at the time of retrieval are the main ones proposed. As noted by Mather et al. (2003), attentional focus at encoding may provide a partial explanation, but their finding that new items are attributed in a choice-supportive manner points to the importance of the retrieval stage, as that is when source attribution takes place. When the source is not clearly remembered, the knowledge (or belief) of what choice one made may be used as an aid to infer the most likely source (Mather and Johnson, 2000). Indeed, Henkel and Mather (2007) found that the belief – at the time of retrieval – that one had made a particular choice, was sufficient to yield choice-supportive memory even when that belief was in fact incorrect.

Explanations focused on emotional and motivational factors point to the influence of emotional goals: the desire to feel that one has made the right choice and that the chosen option is superior to the foregone one may reduce regret and promote well-being (e.g., Taylor and Brown, 1988; Kunda, 1990). Mather et al. (2003) suggested that the belief and desire to have made the right decision provide a likely explanation for choice-supportiveness. Cognitive factors offer an alternative or complementary explanation. As the processing of stereotype inconsistent information has been found to be more cognitively demanding than that of stereotype consistent data (e.g., McIntyre and Craik, 1987; Henkel et al., 1998), it may follow that any information inconsistent with the final choice may require more elaboration and cognitive effort. If such processing is rendered more difficult by age or cognitive capacity, there may be an increased reliance on feelings.

### Fact Distortion

As pointed out by Svenson et al. (2009), most research on memory distortion after decision making has focused on the subjective evaluation of the relevant facts rather than the recollection of the quantitative facts themselves. The focus of the three experiments covered in their paper and in three of the experiments in DeKay et al. (2014) was therefore the memory of the facts provided to the participants when asking them to make a choice between two options.

Similar to the studies in the *misattribution* category, both Svenson et al. (2009) and DeKay et al. (2014) found systematic misremembering favoring the chosen option and downgrading the foregone option. However, DeKay et al. (2014) looked at memory misremembering resulting from predecisional distortion of options based on whether they were leading or trailing alternatives early on in the decision process, whereas Svenson et al. (2009) were interested in the effect of choice on memory. Interestingly, all the experiments in the DeKay et al. (2014) paper and two out of three of those in the Svenson et al. (2009) paper found that most of this distortion stemmed from upgrading the leader or chosen alternative, and only one (Study 3, Svenson et al., 2009) that downgrading the foregone alternative contributed to most of the distortion. In Experiment 4 of the DeKay et al. (2014) paper, downgrading the trailer did not even reach significance. The general trend in these six experiments is thus that bolstering the favored option is the main contributor to this kind of memory distortion. However, the proposed mechanisms behind these effects can be considerably different and point to the importance of methodology when trying to discern underlying factors.

DeKay et al. (2014) found that participants’ predecisional distortion of attributes correlated with their postdecision memory of them and cannot be attributed to response bias or any processes occurring after the choice. The suggested explanation is instead that new information is subjectively encoded as superior relative to its true value if it belongs to the currently leading option, and as inferior relative to its true value if it belongs to the trailer. This bias during encoding then would cause the memory distortion observed during the recall of facts. The authors also concluded that the errors appear to stem from biases in the mental representation of the facts rather than from the judged importance of the information. DeKay et al. (2014) argued, following the outcome of specific control analyses in their studies, that response biases and inferences made from one’s choice are unlikely to explain choice-supportive memories of their participants. Thus, the authors allege that it is the initial mental representation of the information rather than any later processes that gives rise to the distortions in memory.

Svenson et al. (2009) on the other hand, argued that the systematic self-serving fact distortion that they observe in their three experiments is more likely to have arisen in the postdecision stage, as the decisions were made with the information externally available. Thus, at the moment the decision was made, the facts could not be misperceived. The observed memory distortions were predicted by the differentiation-consolidation theory (e.g., Svenson, 1992), according to which decision making is a process of differentiation between the alternatives before deciding and then of consolidation of the decision once made. Thus, the delay between the decision and the recall of that decision would allow the strengthening (consolidation) of the decision and the decision maker’s confidence in it by means of increased choice-supportive memory. According to the differentiation-consolidation theory, fact distortion may occur either before or after a decision has been made and thus potentially both during encoding and during consolidation and it can be related both to cognitive factors (schema/gestalt-related processing) and to emotion-related factors (regret avoidance).

More studies investigating fact distortion and the time course of the observed effects in different scenarios and using a variety of methods will be useful to better understand when choice-supportive fact distortion takes place in different circumstances. It is also important to point out that fact distortion can be investigated only with free recall (and cued recall) and not with recognition, which represents the test that has been used in the great majority of studies on choice-supportive misremembering.

### False Memory

The third type of self-serving memory distortion proposed in our taxonomy is false memory; when attributes or facts not previously presented are ‘remembered.’ Most often, false memories have been studied as a consequence of misinformation from the experimenter (e.g., Ayers and Reder, 1998; Bernstein et al., 2005). The wider concept of false memory, however, has also been observed in studies where participants are asked to make a judgment after attentive consideration of evidence (e.g., Lindholm et al., 2014).

Studies offering evidence for choice-supportive false memories are reported by some of the papers fulfilling the inclusion criteria for this review and using source recognition when testing the memory of the options (e.g., Mather and Johnson, 2000; Mather et al., 2003; Henkel and Mather, 2007). In those studies, the fact that some new features presented in the recognition test were recognized as old can be interpreted as indirect evidence of false memory, although other explanations are possible (e.g., the use of a more inference-based decision strategy for the ‘new’ items, perceived as less accessible).

None of the reviewed studies assessed false memory using free or cued recall. In particular, free recall tests will be highly informative in that they may provide less ambiguous and indirect information on the occurrence of a false memory than the just-mentioned source recognition studies. In future research, it would be useful to investigate the conditions needed for false memories to arise even without misinformation and when memory is assessed using free or cued recall (e.g., long delay). Moreover, considering that the memory literature has highlighted sizable individual differences in proneness to false memories (e.g., Winograd et al., 1998; Zhu et al., 2010), it would be interesting to consider the role of individual differences in choice-supportive false memories.

The papers reviewed do not discuss the specific mechanisms behind false memories directly, as none of them focused exclusively on this type of misremembering. Constructive or schema-based explanations (Loftus, 1995), fuzzy trace theory (Reyna and Brainerd, 1995), and the source monitoring framework (Johnson et al., 1993) are three of the main theories attempting to explain the mechanisms behind false memory and they can be applied also in the context of choice-supportive false memories. Constructive or schema-based explanations assume that false memories originate from semantic integration and inferences that can change memory traces or simply produce competing and thus interfering traces (Loftus et al., 1978). Fuzzy trace theory stresses the distinction between verbatim and gist memory traces, where verbatim traces focus on precise details and gist traces on core meaning. These two types of traces arise in parallel, but verbatim traces are more susceptible to interference and the negative effects of increased delay rather than gist traces. Thus, false memories may arise when the gist affects remembering of verbatim information, or when verbatim memories from different sources are confused with one another. The source monitoring framework, on the other hand, stipulates that false memories are caused by thoughts, images and feelings from one source being mistakenly attributed to another source.

In the context of our taxonomy false memories may consist in the production of an entirely new attribute with its values. This kind of phenomenon is more difficult to explain by making reference to wrong source attribution and easier to explain referring to constructive semantic processes or gist-based influences, assuming that the new attribute and their values are semantically consistent with the choice context and the overall attractiveness of choice options. However, choice-supportive false memories may be alternatively considered as failures of reality monitoring (i.e., the inability to discriminate between internal and external sources of information), although it would remain unexplained how these memories are initially formed. We will come back later to the theories of false memory in the general discussion on the explanations of the various types of misremembering in our taxonomy.

An understanding of the false memory phenomenon is important in the context of choice-supportive memory not only because it is one main type of misremembering, but also because the false memory literature shows that both actual events and falsely remembered events can affect our future attitudes and possibly our future decisions. For example, implanting false memories about loving asparagus the first time they were tried led participants to appreciate the food more and be willing to pay a higher sum for it (Laney et al., 2008), whereas false memories about becoming ill after eating a particular food (Bernstein and Loftus, 2009) or drinking a particular alcoholic beverage (Clifasefi et al., 2013) diminished their liking of it, although consolidated food-related behavior seems difficult to change (Bernstein and Loftus, 2009). In line with these findings, Henkel and Mather (2007) found that memory was affected by the choice the participants thought they had made rather than the one they had actually made.

### Selective Forgetting

The final type of misremembering in our taxonomy is what we have labeled ‘selective forgetting’: when the negative features of the chosen option or the positive features of the foregone option are selectively forgotten. Mather et al. (2000) observed not only choice-supportive misattribution, but also choice-supportive recognition. That is, participants were more likely to recognize positive features of the selected option than positive features of the foregone option, thus showing selective forgetting of the latter ones. Choice-supportive recognition, however, was observed only for positive and not for negative features and not in all the scenarios tested. Thus, in some scenarios, “which option participants selected affected which positive items but not which negative items they recognized as old” (Mather et al., 2000, p. 136).

Depping and Freund (2013) primarily investigated selective forgetting (rather than misattribution or fact distortion). The main conclusion of their studies was that processing of decision-relevant information promotes a stronger focus on negative information in older adults and older adults remember more negative information in choice contexts. However, in their studies, Depping and Freund did not observe significant choice-supportive selective forgetting.

Other studies, not reviewed here because not specifically concerned with choice-supportive memory, suggest that making a choice may produce selective forgetting (or remembering). For instance, Biehal and Chakravarti (1982) contrasted memory for options after choice vs. directed learning of the same information. They observed that memory for chosen options had a similar level of accuracy as memory under directed learning, while accuracy of memory for rejected brands was poorer. The possible implication is that decision makers focus more on the chosen option and on the more choice-relevant information and this may have consequences for subsequent memory, both in terms of better remembering of the chosen option and, possibly, in terms of choice-supportive remembering. However, although there is some evidence that choice-supportive selective forgetting may take place, as we have seen, the evidence is very limited and more studies are needed.

For what concerns potential explanations, existing research, together with research in related topics like confirmation bias (Nickerson, 1998) and incidental and motivated forgetting (Bäuml, 2008; Anderson and Huddleston, 2012; Anderson and Hanslmayr, 2014), suggest that both biased encoding and biased retrieval processes may contribute to the phenomenon, possibly together with suppression of information not supportive of the chosen option and retrieval-based strengthening of supportive information. Interestingly, related research seems to suggest a more important role for encoding-related processes in this kind of distortion as compared, for instance, to more retrieval-based phenomena (like false memories). However, direct research on the processes underlying choice-supportive selective forgetting is lacking and new studies on this topic are needed.

## General Discussion and Conclusion

### Summary of the Findings

In this review, we have presented a novel taxonomy of choice-supportive misremembering after decision making and reviewed papers where the participants make a deliberate choice between options described by multiple attributes and their memory of those attributes is then tested. Our taxonomy represents a theoretically and empirically derived classification of the main types of misremembering after choice: misattribution, fact distortion, false memory, and selective forgetting.

Misattribution is by far the most frequently investigated phenomenon and there is good evidence for it when source recognition tests are used. Indeed, the reviewed evidence seems robust and manifests itself primarily as biased attribution of positive features to the chosen option rather than negative items to the foregone option. Conversely, fact distortion has rarely been investigated, and merits further research, and so does selective forgetting (for which only weak evidence exists). Although some studies provided some evidence compatible with the existence of these two latter types of distortions, there is clearly not sufficient research to date to draw solid conclusions as to the extent of choice-supportive memory in these categories. Therefore, further studies are needed to clarify whether these phenomena are robust, especially when memory is assessed through free or cued recall. The evidence for choice-supportive false memories after decision making is also meager and obtained mainly with a recognition paradigm, which may complicate the interpretation of the findings due to potential alternative explanations.

### Proposed Explanations

Most of the papers where choice-supportive memory was observed do not delve deeply into the proposed mechanisms and explanations behind the phenomenon, but several theories can account for the various types of decision-related misremembering observed. The proposed explanations of the effects can be broadly classified in ‘cognitive’ vs. ‘affective,’ with some accounts making reference to both aspects. At the moment, the relative roles of cognition and emotion are not entirely clear. Neither have the specific processes behind choice-supportive misremembering been fully ascertained, even if some studies have provided preliminary evidence.

From a cognitive perspective, Festinger’s cognitive dissonance theory (Festinger, 1957) generally predicts that healthy adults seek to avoid holding conflicting beliefs or values, and thus tend to distort them in a manner that reduces that dissonance. This would imply that after making a choice one’s memory of the options would be distorted in a manner that would diminish any conflict and the choice would be remembered as more consistent (e.g., Brehm, 1956). The process of reducing conflict could be instrumental in reaching a decision, and continue once it has been made. The result would be choice-supportive memory. A cognitive account of choice-supportive misremembering can also be provided by the fuzzy trace theory (Reyna and Brainerd, 1995), suggesting that memory processes can produce verbatim or gist representations, with the former focusing on specific details and the latter on the core meaning of experiences. When a choice has to be made, gist is more important and better remembered than the precise details and the individual may therefore remember mainly that one alternative was superior enough to be chosen, and this general idea of superiority may then bias memory toward choice-supportiveness. Likewise, schema-driven or constructive processing (e.g., Sulin and Dooling, 1974; Loftus, 1995; see also Dooling and Christiaansen, 1977) would imply that memory would be distorted in agreement with the mental representation of the choice made (i.e., the chosen option is better than the alternative one and thus it was selected). Finally, the source monitoring framework (Johnson et al., 1993; Mitchell and Johnson, 2000, 2009; Johnson, 2006) would explain choice-supportive misremembering either in terms of confusion between different sources (options) for a retrieved item or in terms of a failure of discrimination between internal and external sources of information (depending on the type of misremembering).

Among these theories, source monitoring seems to be naturally and directly applicable to the misattribution category in our taxonomy (as a case of source attribution error), but it is less directly applicable to selective forgetting, false memory, and fact distortion. This does not mean that the theory cannot explain these effects, because they could be considered as the result of confusion between internal and external sources of information, but their explanation would require additional assumptions and specification. In particular, source monitoring needs additional major assumptions to cover selective forgetting. Moreover, the theory should also be able to explain how false memories are generated before being confused with the reality and how attribute values are distorted before being associated with real options, and why wrong attributions tend to boost the chosen option and demote the foregone one (e.g., perhaps due to the knowledge of one owns choice or related beliefs). Furthermore, bringing knowledge- or belief-related assumptions into the theory would blur the distinction between the source monitoring framework and the constructive/schema-related theories. These latter theories, as well as fuzzy-trace theory, have complementary strengths/weaknesses: they seem more able to explain choice-supportive false memory, fact distortion, and selective forgetting, due to postulated semantic/knowledge or gist-based influences on encoding and/or retrieval processes, and perhaps less directly able to explain choice-supportive misattribution. Neither can it be excluded that different mechanisms may explain different kinds of misremembering in out taxonomy. Additionally, it is also important to remember that other specific processes may even be involved in specific cases, like inhibition of non-supportive information or retrieval-based strengthening of supportive information in the case of selective forgetting/remembering (e.g., Bäuml, 2008).

From an affective perspective, memory may be choice-supportive as an implicit means to enhance positivity about oneself and one’s decision making via emotion regulation (Mather and Carstensen, 2005). Indeed, socioemotional selectivity theory underlines the individual’s adaptations to her or his life course, with the reduction in time horizons strengthening the motivation to preserve emotional balance (vs. knowledge-related goals), and leading to a greater focus on emotion regulation and positive aspects of life (e.g., Carstensen, 2006). These changes are thought to affect attention and memory processes in a way that promotes the maintenance of a positive emotional state also via choice-supportive misremembering. This would be in line with the notion of self-protecting memory in the field of autobiographical memories: the motivationally driven pursuit of a positive self-definition taking precedence over accuracy and truthfulness (see e.g., Sedikides et al., 2004; see also Campbell and Sedikides, 1999; Sedikides and Green, 2000; Tesser, 2001). An explanation referring both to cognitive and to emotional factors is provided by the differentiation-consolidation theory (Svenson, 2003). The theory holds that, once the differentiation process needed to reach a decision has been completed, “postdecision processes (called consolidation) work in support of the chosen alternative to maintain this alternative as the preferred gestalt separated from the non-chosen alternative, but also to protect the decision against poor outcomes, regrets, and so on” (p. 291). This suggests the intriguing possibility that multiple and diverse determinants underlie choice-supportive misremembering.

One of the major questions still open is therefore whether a higher degree of choice-supportive memory is better explained by higher degree of emotion regulation or whether it instead reflects more reliance on schema-driven, gist-based processing, or on error-prone source monitoring. Some of the evidence for the affective explanation of choice-supportive memory comes from studies comparing older and younger adults, starting from the assumption that older adults, due to their greater effort in maintaining a positive emotional balance due to age-related changes in high level goals (Mather and Carstensen, 2005; Carstensen, 2006), would show a greater degree of choice-supportive memory distortion than younger adults. Indeed, although a difference was found in one study (e.g., Mather and Johnson, 2000), as we have seen, the evidence is generally negative. In the study of Mather and Johnson (2000), the results of the ‘affective review’ condition in younger adults also point to the role of socio-emotional factors.^2^ However, the negative relation between control measures and choice-supportive distortion in older participants is not in agreement with the general statement that control abilities are needed to ensure emotion-regulation success (Mather and Carstensen, 2005; Mather and Knight, 2005), suggesting to the need for clarification of the role of cognitive control in different kinds of positivity biases. A possibility is that less effective active encoding and recollection processes, together with emotion-related factors, might contribute to older adults’ stronger positivity bias for past choices (Mather and Johnson, 2000) – assuming that this age-related exacerbation of the bias exists – with the decline of executive control processes playing a significant role (Del Missier et al., 2012, 2015).

Another issue that would deserve more investigation is to elucidate more precisely the possible interplay between emotional and cognitive factors in determining choice-supportive misremembering and to provide more direct evidence for the proposed relations. While some approaches, like the socio-emotional one, seem to imply that affective and motivational drivers affect memory of choice options via cognitive mechanisms like those underlying biased source attribution (Mather and Johnson, 2000; Mather and Carstensen, 2005; Mather and Knight, 2005), there is no sufficient evidence, at the moment, to support empirically a more distal role of affect and a more proximal one of cognition. It may well be that cognition contributes to choice-supportive monitoring beyond emotion, as cognitive theories of false memory seem to suggest. And, as we have just discussed, more studies are also needed to better appraise the specific cognitive and emotional mechanisms involved and the time course of their potential interaction. Clearly, given the mixed results of the studies, research investigating more directly the paramount issue of the processes underlying the choice-supportive misremembering is needed, both to shed light on the respective contribution of cognitive and emotional factors and to clarify what kinds of cognitive (e.g., cognitive control, attention, episodic encoding and/or retrieval) and emotional processes (e.g., implicit or explicit emotion regulation, regret avoidance, goal setting) are involved.

### Limitations and Future Directions

Our review did not cover all the factors that could potentially influence memory after choice. Given our necessarily restrictive eligibility criteria, we left out studies based on information provision after choice (e.g., hindsight bias and misinformation effects) and investigations based on the self-selection of information before choice. Another limitation is represented by the fact that the reviewed studies used a variety of methods and materials, which may have affected the specific results obtained in specific circumstances. For this reason, we included an analysis of potentially moderating factors, as a first step toward a systematic experimental appraisal of their role.

Our extensive search for published and unpublished studies on misremembering after a deliberate choice between options described by multiple attributes yielded a surprisingly low amount of papers, and pointed to several gaps in the literature. A fundamental question that remains to be answered is whether choice-supportive memory can be shown to be a robust phenomenon even in studies not focusing on misattribution and not testing only recognition memory. More research is also needed both on the temporal aspect of distortions (at encoding, during memory consolidation or retention, at retrieval), on the relative contribution and type of cognitive and socio-emotional processes involved, and on their interactions. This implies setting up studies specifically targeting the time-course of choice-related misremembering and their underlying processes, using both experimental and individual-difference approaches, eventually together with neuroimaging investigations capable of highlighting the cognitive and affective development of processing. It would also be interesting to explore choice-supportive memory in clinical populations (e.g., in patients with damage to the orbitofrontal cortex vs. the dorsolateral cortex vs. different areas in the temporal lobe, and in patients with autism spectrum disorder or alexithymia) to help unveiling the processes involved, and in children and adolescents to elucidate the developmental aspect.

Research on individual differences would also be useful to elucidate the relationships between individual differences in cognition, motivation, emotion and choice-supportive memory, as this could provide useful information about potential explanations of choice-supportive misremembering. For example, if individual differences in need for closure and rumination or regret were found to correlate with the degree of choice-supportiveness, this would lend support to the importance of motivational or emotional factors. Conversely, correlations between the degree of choice-supportiveness and the effectiveness of recollection measures would support a more cognitive account.

As a final issue, it would be useful to understand to what extent choice-supportive memory can lead to suboptimal decisions in repeated (or related/similar) future memory-based or mixed decisions (Chen and Zhang, 2003) and how it is connected to emotional balance, self-esteem and life satisfaction, in order to properly weigh the relative costs and benefits of choice-supportive misremembering. Just as it is important to assess the behavioral consequences of false memories (e.g., Bernstein et al., 2005; Laney et al., 2008), a better understanding of the behavioral influence of choice-supportive misremembering over time would be fruitful. Along this line of investigation, individual differences in proneness to different types of choice-supportive misremembering could also be examined in relation to personality variables to appraise when a normal and even adaptive degree of self-deception turns into a dangerous and delusional alteration of reality.

## Author Contributions

ML: Main theoretical contribution in devising the taxonomy and the review; paper search and collection; main contribution in analysis and writing. MV and TM: Theoretical contribution in refining the taxonomy and the review; contribution in the writing stage. FD: Main theoretical contribution in devising the taxonomy and the review; contribution in analysis and writing.

## Conflict of Interest Statement

The authors declare that the research was conducted in the absence of any commercial or financial relationships that could be construed as a potential conflict of interest.

## Footnotes

1 The same misremembering classification can be applied in a straightforward way to attribute values that are not just perceived as positive or negative, but simply as better or worse along a common evaluation dimension. For instance, assuming that a lower price is preferable to a higher price for a product, but that a decision maker has chosen between two options the product with the higher price due to its (other) better features, choice-supportive misattribution would imply switching the association between options and prices when remembering them. Choice-supportive fact distortion would imply remembering a price lower than the true one for the chosen option and/or a higher one for the foregone option. If price had not been presented at all, remembering that the chosen option had a lower price than the alternative one would represent a case of choice-supportive false memory. Finally, forgetting the price would represent an example of choice-supportive selective forgetting.

2 Rather surprisingly, Mather and Johnson (2000) failed to observe such distortion in the control condition in younger adults.
